# Return to work after surgery for degenerative cervical myelopathy: a nationwide registry-based observational study

**DOI:** 10.1007/s00701-023-05521-w

**Published:** 2023-02-16

**Authors:** Vetle Vangen Lønne, Sozaburo Hara, Sasha Gulati, Lene Aasdahl, Øyvind Salvesen, Øystein Petter Nygaard, Tore Solberg, Karen Walseth Hara

**Affiliations:** 1 Department of Neurosurgery, St. Olav’s University Hospital, Trondheim, Norway; 2 Department of Neuromedicine and Movement Science, Norwegian University of Science and Technology, 7006 Trondheim, Norway; 3 National Advisory Board for Spinal Surgery, St. Olav’s University Hospital, Trondheim, Norway; 4 Department of Public Health and Nursing, Norwegian University of Science and Technology, Trondheim, Norway; 5 Unicare Helsefort Rehabilitation Centre, Rissa, Hasselvika, Norway; 6 Unit for Applied Clinical Research, Norwegian University of Science and Technology, Trondheim, Norway; 7 Department of Neurosurgery, University Hospital of Northern Norway, Tromsø, Norway; 8 Norwegian Registry for Spine Surgery (NORspine), Tromsø, Norway; 9 NAV Advisory Service for Trøndelag, Trøndelag, Norway

**Keywords:** Return to work, Degenerative cervical myelopathy, Spine surgery, Cervical

## Abstract

**Background:**

Few studies of high quality exist on return to work (RTW) rate after surgery for degenerative cervical myelopathy (DCM). This study aims to examine the RTW rate in patients undergoing surgery for DCM.

**Methods:**

Nationwide prospectively collected data were obtained from the Norwegian Registry for Spine Surgery and the Norwegian Labour and Welfare Administration. The primary outcome was return to work, defined as being at work at a given time postoperatively without any medical income-compensation benefits. Secondary endpoints included the neck disability index (NDI) and quality of life measured by EuroQol-5D (EQ-5D).

**Results:**

Among 439 patients operated for DCM between 2012 and 2018, 20% of the patients received a medical income-compensation benefit one year before surgery. This number increased steadily towards the operation at which timepoint 100% received benefits. By 12 months after surgery, 65% had returned to work. By 36 months, 75% had returned to work. Patients that returned to work were more likely to be non-smokers and to have a college education. They had less comorbidity, more were without benefit 1-year pre-surgery, and significantly more patients were employed at operation date. Average days of sick leave in the year before surgery were significantly less in the RTW group, and they had a significantly lower baseline NDI and EQ-5D All PROMs reached statistical significance at 12 months, in favor of the group that achieved RTW.

**Conclusion:**

At 12 months following surgery, 65% had returned to work. At the end of the 36-month follow-up period, 75% had returned to work, 5% less than the working percentage in the beginning of the follow-up period. This study demonstrates that a large percentage of patients return to work after surgical treatment for DCM.

## Introduction

Degenerative cervical myelopathy (DCM) is a progressive spine disorder and the most common cause of spinal cord impairment in adults over 55 years [18, 23, 26, 37]. Degenerative changes in the cervical spine such as disk herniation, ligament hypertrophy or ossification, and osteophyte formation may lead to compression and dysfunction of the spinal cord [9, 26]. Symptoms of DCM include pain and stiffness in the neck, pain and numbness in limbs, poor coordination, imbalance, frequent falls, loss of dexterity, and incontinence [4, 38]. Several symptoms of DCM are non-specific and subtle and overlap with other neurological conditions, which makes early diagnosis a challenge. Lack of awareness and incomplete neurological assessment can also delay diagnosis, which may increase patients’ risk of developing life-long disability and impaired quality of life [3, 27, 36].

Neck and back pain are leading causes of absence from work [24, 42]. Recent studies examining outcomes after surgery for DCM found significant improvement for both mild, moderate, and severe DCM measured with several different patient-reported outcome measures (PROMs) [12, 17]. Due to the high relevance for the individual and society, return to work (RTW) has become an important outcome measure in recent years [5]. Few studies of high quality exist on RTW after surgery for DCM [13, 20]. As the working population continues to grow older and wishes to stay active and working, knowledge about RTW for patients with DCM is paramount. Further, there are few established predictors for RTW after undergoing surgery for DCM. This study aims to examine the RTW rate in patients undergoing decompressive surgery for DCM.

## Materials and methods

Reporting is consistent with the Strengthening The Reporting of Observational Studies in Epidemiology (STROBE) statement [40]. The Regional Committee for Medical Research Ethics in Central Norway approved the study (No. 2016/840), and all participants provided written informed consent. Data from the Norwegian Registry for Spine Surgery (NORspine) and the Norwegian Labour and Welfare Administration (NAV) were linked individually for each participant. This research group recently published a study examining RTW after surgery for cervical radiculopathy, using a similar approach [13].

### Study population

We collected data from patients who underwent decompressive surgery for DCM between January 1, 2012, and June 15, 2018. Patients were considered eligible if they were between the age of 18 to 60 years old, diagnosed with cervical myelopathy, included in NORspine, and received a temporary medical benefit (any grade of sickness benefit or work assessment allowance) on the day of surgery. Patients who did not receive a temporary benefit on the day of surgery (i.e., students, homemakers, retired, recipients of full disability benefit) were excluded. Patients over the age of 60 were excluded, as retirement pension in Norway can be taken out at the age of 62 at the earliest, and we wanted to examine a group that were in working age following surgery.

### Surgical procedures

All patients underwent decompressive surgery of the cervical spine. The surgical approach, the number of operated levels, and the use and type of instrumentation were determined at the surgeons’ discretion.

### NORspine

Norwegian Registry for Spine Surgery (NORspine) is a comprehensive clinical registry for research and quality control [25]. It provides data on demographics, lifestyle, comorbidity, diagnoses, clinical and radiological findings, surgical procedures, and complications, as well as PROMs before and after spinal surgery [25, 34]. Currently, all 40 centers performing lumbar spine surgery in Norway report to NORspine, and approximately 81% of patients who undergo surgery on the cervical spine are included in NORspine. The inclusion rate for DCM surgery is probably higher as these procedures typically are scheduled and rarely performed as emergency surgery [35]. NORspine participation was not a requirement for patients to gain access to treatment or for a provider to be eligible for reimbursement and payment. On admission for surgery (baseline), the patients completed the self-administered baseline questionnaire. During the hospital stay, the surgeon recorded relevant data using a standard registration form. Follow-up questionnaires were distributed to patients by regular mail at three months and one year after surgery, completed at home by the patients and returned. The patients who did not respond received one reminder with a new copy of the questionnaire. The patients completed all the questionnaires without any assistance from the surgeon or other staff from the treating hospital.

### Norwegian Labour and Welfare Administration (NAV)

Norway has a comprehensive national insurance scheme administered by the Norwegian Labour and Welfare Service (NAV). Economic loss due to sickness and injury is generously compensated. Medical benefits issued by NAV are summarized as follows:

- Sickness benefit (temporary and short-term: partial or full): Every member of the society who has worked in Norway continuously for six weeks is entitled to a sickness benefit for the first 12 months of sick leave. This compensates previous salary with 100% coverage, with some limitations regarding size of the salary.

- Work assessment allowance (temporary and long-term: partial or full): Persons who cannot resume work after this period and are under ongoing medical treatment or with a possibility of improving may apply for a benefit termed work assessment allowance for the next 36 months. This compensates on average about 66% of the income. In addition, persons may be entitled to work assessment allowance without working experience if their ability to work is impaired due to illness or injury (e.g., students, handicapped, refugees with health problems). Sickness benefits and work assessment allowance are mutually exclusive.
- Disability benefit: Disability benefits may be warranted for those permanently disabled to work, either partially or fully. Patients with partial disability benefits are considered actively working, albeit with a reduced work capacity.

### Primary outcome measure

#### RTW

Our primary outcome was return to work (RTW), defined as being at work at a given time postoperatively without a medical income-compensation benefit from NAV. We calculated the grades of received benefits (partial or full sick leave, partial or full work assessment allowance, partial or full disability benefit) for each day from 1 year before to 3 years after surgery. The benefits were then grouped into five categories: no medical benefit, partial medical benefit of any kind, full sickness benefit, full work assessment allowance, and full disability benefit. We then examined the data on a group level and explored the trends in sick leave and RTW for our patient group.

### Secondary outcome measures

#### PROMs

The neck disability index (NDI) is a self-rated questionnaire developed for patients with neck disabilities [16]. The questionnaire is composed of 10 items: 7 related to activities of daily living, 2 to pain, and 1 to concentration. The sum of the 10 items is recalculated into a percentage NDI score from 0 to 100 (no to maximum disability). The minimal clinically important change (MCIC) is 4.3 percentage points [21, 22, 43].

The European myelopathy score (EMS) is a questionnaire with 5 subscores designed to evaluate the 4 major neural systems, the impairment of which contributes to the clinical picture of DCM: (a) the upper motor neuron with signs of spasticity, bladder and bowel disturbances; (b) the lower motor neuron with impairment of hand function; (c) the posterior roots with upper limb radicular deficits and paresthesias; and (d) the posterior columns with proprioceptive sensory loss, disturbed coordination, and ataxia [2, 39]. The total score ranges between 5 and 18, and the lower the score, the more severe the deficits. Scores ≥13 were classified as mild DCM and scores between 5 and 12 points were classified as moderate-to-severe DCM [39]. There is no consensus on the MCIC for EMS, but even a small change in severe DCM might be considered important in daily function.

Changes in health-related quality of life were measured with EQ-5D [32]. An index value for health status is generated for each patient. Scores range from − 0.6 to 1, in which 1 corresponds to perfect health. Effect size estimations were used to evaluate the magnitude of changes [6]. EQ-5D also contains a vertical visual analog scale, ranging from 0 to 100 (lower scores indicate poorer health).

Headache, and neck and arm pain were assessed with a numeric rating scale (NRS) from 0 to 10, with response options ranging from 0 (no pain) to 10 (worst imaginable pain). The MCIC for NRS is approximately 1.5 points [6].

The Global Perceived Effect (GPE) scale has seven response categories: (1) complete recovery, (2) much better, (3) slightly better, (4) unchanged, (5) slightly worse, (6) much worse, and (7) worse than ever [19].

### Statistics

Statistical analyses were performed with STATA 16.1 and 17.0 (StataCorp., College Station, TX) and SPSS version 27 (IBM Corporation, IL). The population was divided into two groups, the group that successfully returned to work at 2 years after surgery and the group that did not. We compared the groups for the available variables using a two-sample *t*-test for the continuous variables and Pearson’s *χ*^2^ test for the categorical variables.

Logistic regression analyses were performed with “achieved RTW two years after surgery” as the dependent variable. Variables with a *p* value < 0.05 in a two-sample *t*-test or Pearson’s *χ*^2^ test were selected for a multivariable regression analysis if also considered clinically relevant. All selected variables were analyzed in one single model, with odds ratios calculated from it.

### Missing data

Patients were excluded if they were younger than 18 or older than 60 years old, or if they did not receive a temporary benefit on the day of operation. When examining all longitudinal data from NAV, we found occasional gaps in longer sick leave periods. If the gaps were 28 days or less, they were replaced with the last registered value under the assumption that the gap was due to a temporary work trial, missing registration, or planned vacation. Gaps longer than 28 days were left untouched and treated as “periods without medical benefit.” Twelve-month PROMs data was used as standard. If 12-month data were missing (due to loss to follow-up), 3-month data were used if available.

## Results

**Fig. 1:**
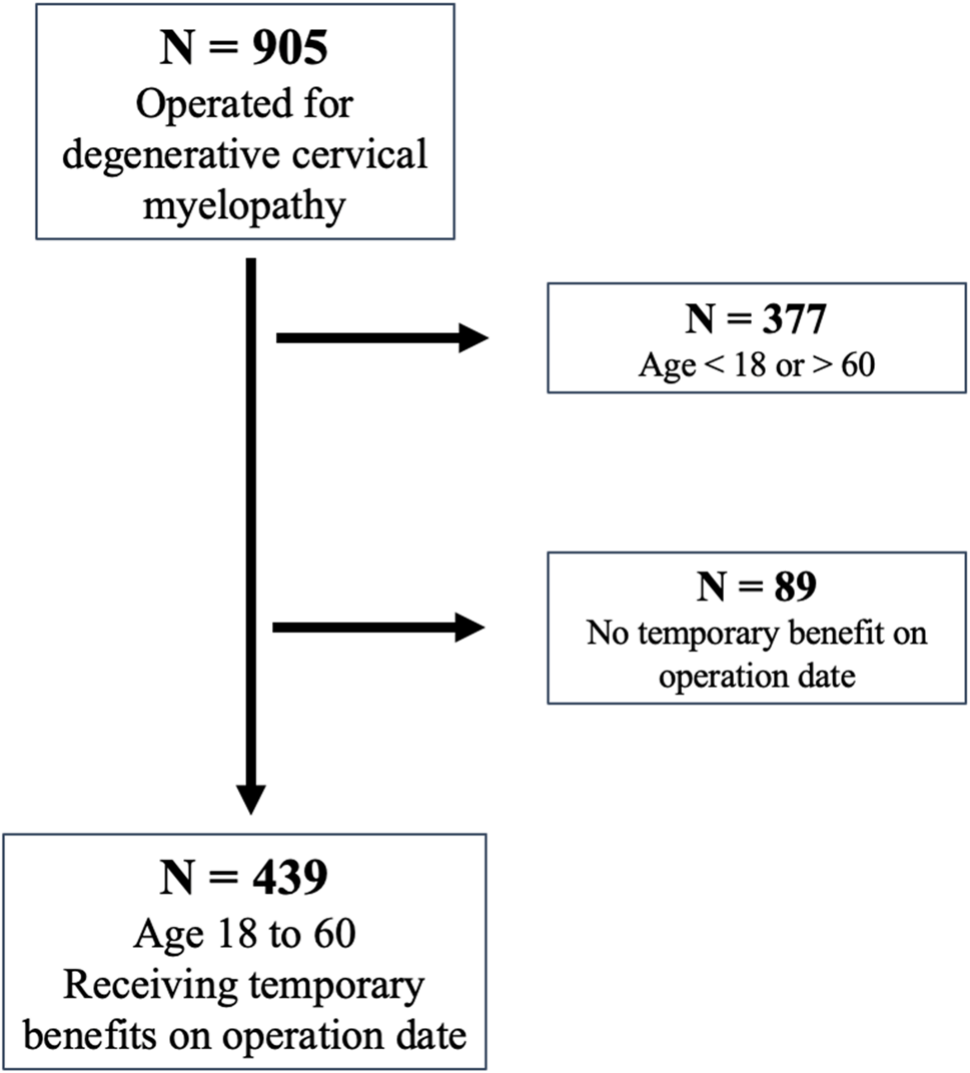
Patients included

Among 906 patients operated for cervical myelopathy, 439 were eligible for our study (Fig. 1). Baseline characteristics are presented in Table 1. Mean age for all included patients was 48 years and 42% were women.

**Table 1:** Demographic and clinical characteristics

Variable	Return to work at 2 years		*p* value
	No, *n* = 130	Yes, *n* = 309	
Mean age at surgery (±SD)	48.8 (7.3)	48.1 (8.1)	0.42
Gender (female)	63 (48.4%)	120 (38.8%)	0.062
Any college education	30 (23.1%)	133 (43%)	<0.001
Employed at operation date	89 (68.4%)	267 (86.4%)	<0.001
Work assessment allowance at operation date	27 (20.8%)	20 (6.5%)	<0.001
No benefit 1-year pre-surgery	88 (67.7%)	263 (85.1%)	<0.001
Smoker	55 (42.3%)	93 (30.1%)	0.025
Obesity (BMI ≥ 30)	43 (33.1%)	81 (26.2%)	0.188
Comorbidity	67 (51.5%)	110 (35.6%)	0.002
Hypertension	15 (11.5%)	28 (9.1%)	0.425
Cardiovascular disease	16 (5.2%)	2 (0.5%)	0.079
Diabetes mellitus	6 (4.6%)	15 (4.9%)	0.915
Chronic neurological disease	2 (1.5%)	6 (1.9%)	0.773
Anxiety/depression	7 (5.4%)	4 (1.3%)	0.012
Rheumatoid arthritis	4 (3.1%)	0 (0%)	0.02
ASA ≥ 3	10 (7.7%)	17 (5.5%)	0.588
Pain > 1 year	29 (22.3%)	66 (21.4%)	0.589
Mild DCM pre-surgery	107 (82.3%)	264 (85.4%)	0.646
Moderate DCM pre-surgery	14 (10.8%)	24 (7.8%)	0.574
Severe DCM pre-surgery	0 (0%)	1 (0.3%)	0.799
Sick days the year before surgery	183.1 (± 128.1)	100.9 (± 116.0)	<0.001
≤ 90	43 (33.1%)	189 (61.2%)	< 0.001
90–180	25 (19.2%)	52 (16.8%)	0.546
180–270	21 (16.2%)	22 (7.1%)	0.004
> 270	41 (31.5%)	46 (14.9%)	< 0.001

### Primary outcome

**Fig. 2:**
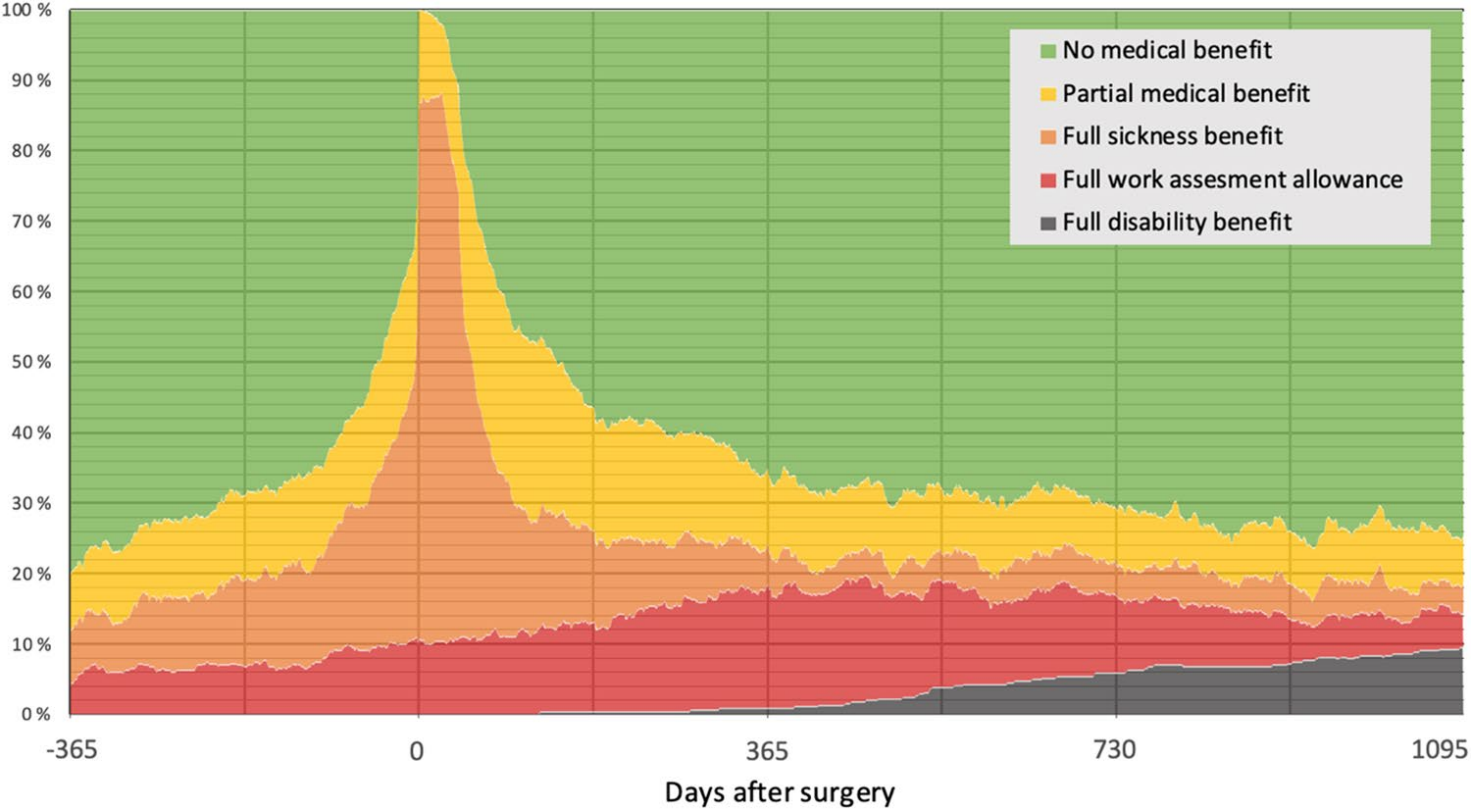
Trends of sick leave benefits from 1 year before to 3 years after surgery

Changes in sick leave benefits throughout the follow-up period are displayed in Fig. 2. One year before surgery, 20% of the patients received any kind of benefit from NAV. This number increased towards the operation date, the main reason being increases in full sickness benefit or partial benefits of any kind. By 1 week before surgery, 66% received some sort of medical benefit. Following surgery, the number of recipients rapidly decreased. The percentage of patients who received full sickness benefit decreased the fastest. By 5 months, 50% had returned to work. The rapid rate of patients returning to work gradually slowed down and flattened out at approximately 12 months, by which time 65% of the patients had returned to work. The percentage of patients receiving full work assessment allowance increases during the first year, peaking at around 12 months. The percentage of patients who received full disability benefit gradually increased from a few months after surgery all the way to the end of the follow-up period, where 10% received full disability benefit. By the end of the follow-up period at 36 months, 75% had returned to work, while 25% still received some sort of benefit. The working percentage decreased by 5%, from 80% at the beginning of the follow-up period to 75% at the end of the follow-up period.

### Secondary outcomes

The patients were divided into two groups: those who achieved RTW at 2 years and those who did not (Table 1). Patients that returned to work were more likely to be non-smokers and to have a college education. They also had less comorbidity overall and were less likely to suffer from anxiety and depression. Work assessment allowance at operation date was more common among the non-RTW group than the RTW group (20.8% vs. 6.5%, *p* < 0.001). Significantly more patients were employed at operation date in the RTW group (86.4% vs. 68.4%, *p* < 0.001), and more were without benefit 1-year pre-surgery (85.1% vs. 67.7%, *p* < 0.001). Average days of sick leave in the year before surgery were significantly less in the RTW group.

**Fig. 3:**
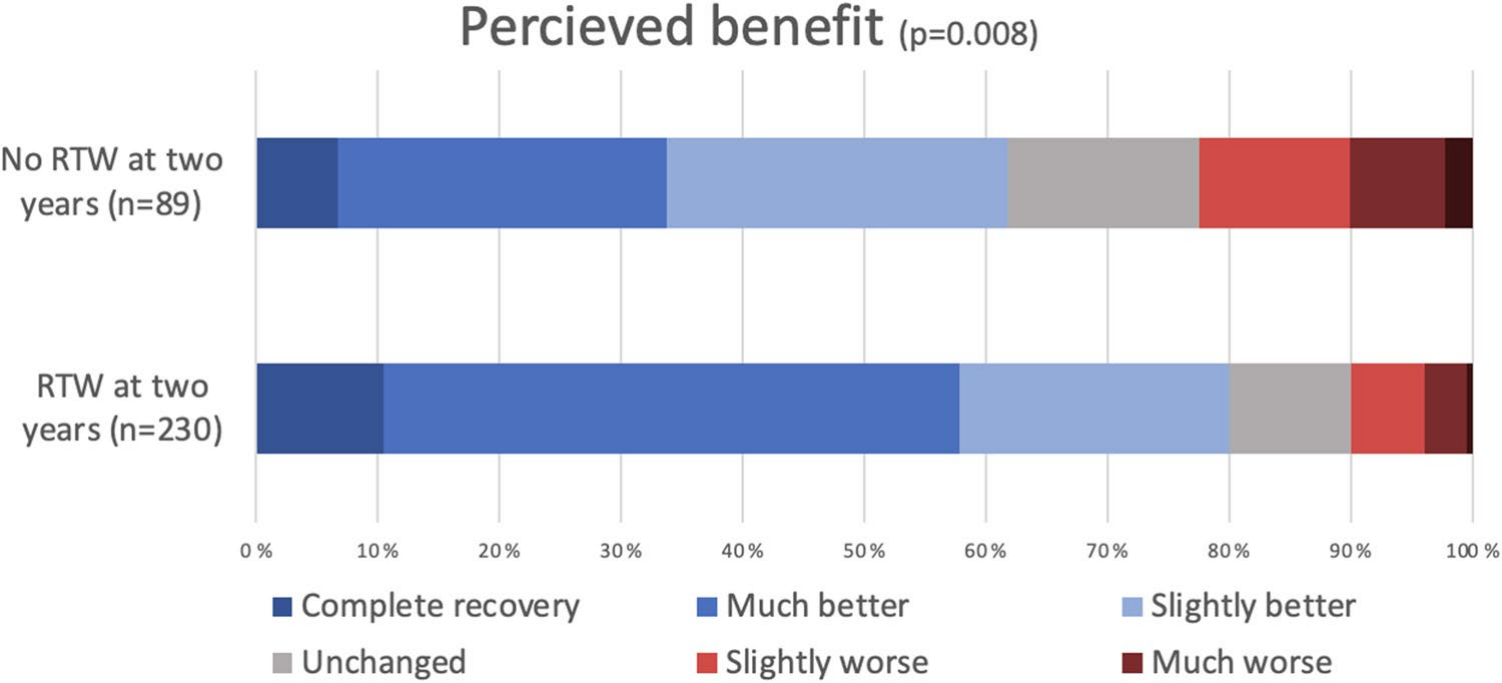
Global perceived effect at one year following surgery for degenerative cervical myelopathy in patients with and without return to work (RTW) at 2 years

The group that achieved RTW at 2 years had a significantly lower average baseline disability measured by NDI (32.5 ± 15.7 vs. 39.8 ± 16.3, *p* < 0.001) and EQ-5D (0.50 ± 0.31 vs. 0.42 ± 0.33, *p* = 0.018) (Table 2). Difference in neck pain and headache at baseline also reached statistical significance (mean NRS neck 4.6 ± 2.9 vs 5.4 ± 2.8, *p* = 0.008, mean NRS headache 4.8 ± 2.8 vs 5.2 ± 2.8, *p* < 0.001). The difference in mean EMS and NRS arm pain did not reach statistical significance at baseline. The difference in perceived benefit according to the GPE scale (presented in Fig. 3) was statistically significant, with 90% in the RTW group reporting “unchanged” perceived benefit or better (vs 78%, *p* = 0.008). All PROMs reached statistical significance at 12 months, in favor of the group that achieved RTW.

**Table 2:** Patient reported outcome measures

Baseline mean values	Returned to work at 2 years		*p* value
	No, *n* = 130	Yes, *n* = 309	
Neck disability index (SD)	39.8 (16.3)	32.5 (15.7)	< 0.001
European myelopathy score (SD)	14.9 (2.0)	15.3 (1.9)	0.057
EQ-5D (SD)	0.42 (0.33)	0.50 (0.31)	0.018
Arm pain numerical rating scale (SD)	5.2 (2.8)	4.8 (2.9)	0.186
Neck pain numerical rating scale (SD)	5.4 (2.8)	4.6 (2.9)	0.008
Headache numerical rating scale (SD)	5.2 (2.8)	4.8 (2.8)	< 0.001
Mean values at 12 months*			
Neck disability index (SD)	33.6 (16.7)	21.3 (17.3)	< 0.001
European myelopathy score (SD)	15.5 (2.0)	16.3 (1.6)	< 0.001
EQ-5D (SD)	0.48 (0.34)	0.71 (0.26)	< 0.001
Arm pain numerical rating scale (SD)	3.9 (2.9)	2.9 (2.7)	0.003
Neck pain numerical rating scale (SD)	4.1 (2.7)	3.0 (2.7)	0.002
Headache numerical rating scale (SD)	3.0 (2.9)	1.98 (2.6)	0.004

*Three-month values were used if 12-month data were not available

The results of the regression analyses are presented in Table 3. College education (OR 3.5, CI 1.76–6.96), less than 90 sick days in the year before surgery (OR 1.99, CI 1.03–3.85) and increasing NRS neck pain (OR 1.28, CI 1.04–1.58) were associated with increased chance of RTW at 2 years. Female sex (OR 0.44, CI 0.23–0.82), increasing NDI (OR 0.95, CI 0.92–0.99), and decreasing EQ-5D (OR 13.1, CI 2.35 – 73.29) were associated with less chance of RTW at 2 years.

**Table 3:** Multivariable logistic regression

Variable	OR	Lower 95% CI	Upper 95% CI	*p* value
Age	0.98	0.94	1.02	0.323
Female sex	0.44	0.23	0.82	0.011
College education	3.50	1.76	6.96	< 0.001
Smoker	0.99	0.51	1.91	0.976
Employed at operation date	1.52	0.60	3.87	0.378
AAP at operation date	1.44	0.45	4.60	0.538
≤ 90 sick days in the year before surgery	1.99	1.03	3.85	0.042
PROMs, mean values at 12 months*				
Neck disability index	0.95	0.92	0.99	0.012
European myelopathy scale	0.83	0.64	1.07	0.141
EQ-5D	13.1	2.35	73.29	0.003
Arm pain numerical rating scale	1.15	0.99	1.35	0.075
Neck pain numerical rating scale	1.28	1.04	1.58	0.023
Headache numerical rating scale	1.12	0.95	1.32	0.189
Global perceived effect scale	0.78	0.58	1.05	0.098

*Three-month values were used if 12-month data were not available

## Discussion

This study examined patterns for returning to work after surgery for DCM as well as predictors for achieving RTW. In total, 50% of the patients returned to work after 5 months, and by 12 months 65% of the patients had returned to work. At the end of the follow-up period at 36 months, 75% had returned to work, 5% less than the working percentage in the beginning of the follow-up period.

In addition to pain, physical disability, and health related quality of life, RTW is increasingly acknowledged as a core outcome measure in spine surgery [5, 41]. Recent studies have shown considerable improved physical function after surgery for DCM which may provide new opportunities to patients who were previously unable to work [8, 12, 17]. Although surgery for DCM results in statistical and clinical meaningful improvement, this is not a guarantee for returning to work. Even so, larger percentage of patients operated on for DCM achieved RTW than in a similar study examining RTW after surgery for cervical radiculopathy [13].

To our knowledge, this is the largest study to date examining RTW after surgery for DCM. Direct comparison with other studies examining RTW-rate after surgery for DCM is challenging [5, 10, 31]. Differences in cohort selections, welfare systems, authors definition of RTW and health care policies in individual countries contributes to this. A study examining RTW for 102 non-retired patients found that 58.8% of the total population achieved RTW at 1 year, while 75.9% of the population who were working pre-surgery achieved RTW [31]. Like our study, working pre-surgery was associated with RTW. This study did, however, include all patients who were considered “non-retired” and had a smaller sample size than our study. A study from 2018 examining RTW after cervical spine surgery found that 82% achieved RTW after three months [5]. They found that patients who achieved RTW were more likely to have higher education, 100% employment, and lower NDI at baseline and three months. However, this study included patients operated on for both cervical myelopathy and radiculopathy and included only patients who were working pre-surgery. A study from 2020 examined RTW, among other outcomes, in 219 patients operated for cervical myelopathy [10]. They found that 96% of patients with mild DCM 100% of patients with moderate DCM and 84% of patients with severe DCM achieved RTW. They did not, however, define RTW clearly in their study, and only reported it as a secondary outcome.

College education, female sex, and less than 90 days of sick leave in the year before surgery, as well as NDI and EQ-5D at 12 months, had the strongest effect on RTW in this study. A study from 2021 examining work ability measured with the Work Ability Index score (WAI) after surgery for cervical radiculopathy found that thoughts of being able to work within the next 6 months, NDI score and work-related neck load explained 59% of the variance in WAI after 2 years of follow-up [29]. A study from 2021 identified occupational profile as a predictor for RTW after surgery for DCM, with manual laborers having the lowest RTW rate [28]. We did not have access to specific occupation in this study, and more research is needed to establish the relationship between occupational factors and RTW rate after surgery for DCM. A study from 2013 examining prognostic factors for RTW in patients with sciatica found that less sciatica bothersomeness at baseline and duration less than 3 months predicted faster RTW [11]. Less than 90 days of sick leave in the year before surgery were associated with higher chances of RTW in our study, indicating that both manageable symptoms and a shorter symptom duration before surgery might contribute to achieving RTW.

In addition to being less likely to have a college education and employment, the patients that did not return to work were more likely to receive some sort of benefit 1 year pre-surgery and had more comorbidity overall. This group might benefit from counseling from primary care providers, employers, or local labor offices. Identifying individuals at risk for not returning to work remains a challenge for all health care providers, and more research is required to help as many as possible return to work after surgery.

### Limitations

This study has several limitations. First, our outcome is based on the medical benefit payment records provided by NAV, and a reduction in benefits is interpreted as an indirect measure of RTW. This method is commonly used in the RTW literature and is likely sufficient in our population [1, 13, 30]. Second, we lack data on social factors, details on occupation, and a detailed psychological profile of each patient. Such information was not available in the data provided to us by NORspine and NAV, but we recommend that they are included in future studies. Third, missing data for PROMs in registry-based studies are a concern. However, a NORspine study showed no difference in outcomes between responders and non-responders [33]. We found no difference in RTW ratios between responders and non-responders in our study, which is consistent with previous studies indicating that non-responders do not bias evaluation of PROMs [7, 14, 15]. Even so, we do not know the exact reasons for non-respondence, and our results must be interpreted with this in mind. Fourth, all patients included in our study were selected for surgery and might not be representative for the total population of DCM patients. NORspine only includes patients that actually undergo surgery, and unfortunately, we do not have any information about patients who did not receive surgical treatment. Patient characteristics, indications, surgical strategies, and medical benefit systems may vary between countries, and results from our study might consequently differ from other clinical settings.

## Conclusion

At 12 months following surgery, 65% had returned to work. At the end of the 36-month follow-up period, 75% had returned to work, 5% less than the working percentage in the beginning of the follow-up period. This study demonstrates that a large percentage of patients return to work after surgical treatment for DCM.

## Abbreviations

DCM: Degenerative cervical myelopathy
RTW: Return to work
NDI: Neck disability index
EMS: European myelopathy scale
EQ-5D: EuroQol-5D
NRS: Numeric rating scale
GPE: Global perceived effect scale
PROMs: Patient-reported outcome measures
NORspine: The Norwegian Registry for Spine Surgery
NAV: The Norwegian Labour and Welfare Administration
